# Response to pneumococcal vaccination in multiple myeloma

**DOI:** 10.1002/cam4.2253

**Published:** 2019-05-30

**Authors:** Loïc Renaud, Susanna Schraen, Guillemette Fouquet, Stephanie Guidez, Hélène Demarquette, Morgane Nudel, Emilie Cayssials, Claire Bories, Charles Herbaux, Thomas Systchenko, Jean‐Luc Faucompré, Antoine Machet, Florence Sabirou, Antony Levy, Arthur Bobin, Valentine Richez, Niels Moya, Cécile Gruchet, Deborah Desmier, Zoe van de Wyngaert, Benjamin Carpentier, Salomon Manier, Thierry Facon, Stephen Harding, Xavier Leleu

**Affiliations:** ^1^ Department of Hematology CHU Lille Lille France; ^2^ Centre de Biologie et Pathologie CHU Lille France; ^3^ Faculté de médecine Hôpital de la Milétrie, and Inserm CIC 1402, CHU Poitiers France; ^4^ Hôpital, CHU Nice France; ^5^ Binding Site Group Ltd Birmingham UK

**Keywords:** ELISA test, multiple myeloma, pneumococcal vaccination, prime boost, serological response

## Abstract

**Background:**

*Streptococcus pneumoniae* infection causes morbidity and mortality in multiple myeloma patients. Pneumococcal vaccination is commonly given to immunocompromised myeloma patients; however response data are sparse. Here, we present longitudinal response data to pneumococcal vaccination in multiple myeloma patients.

**Method:**

Twenty‐eight multiple myeloma patients were included, 25 of whom were newly diagnosed. All the patients received two vaccines Prevnar13® and Pneumo23®. Serotype‐specific IgG was measured by ELISA for all 23 vaccine serotypes at baseline, and then sequentially at different time points postvaccination until treatment ended. Response to vaccination is available for 20 patients. The primary endpoint was the incidence rate of patients who obtained an isotype response serum concentration after vaccination. Secondary endpoints included detailed isotype increase, time to first increase, further assessment of a decreased anti‐pneumococcal serum concentrations following treatment including autologous stem cell transplantation (ASCT), rate of infection with a special attention to pneumococcal infection.

**Results:**

The median age was 66 years and the male to female ratio was 0.6. Anti‐pneumococcal capsular polysaccharide (anti‐PCP23) IgG, IgG2, IgA, and IgM responses were detected within 1 week postvaccination. Response to at least one subtype of antibody was obtained in 85% (n = 17) of patients, for at least two subtypes in 65% (n = 13), for at least three subtypes in 55% (n = 11), and 2 patients responded to all four subtypes. The median increase in the concentration of anti‐PCP23 isotypes was threefold following vaccination, with the highest increase observed when Pneumo23® was given more than 30 days after Prevnar13®. The anti‐pneumococcal geometric mean concentration decreased significantly for all subtypes over time independently of treatment approaches.

**Conclusion:**

Myeloma has the ability to demonstrate a response to pneumococcal vaccine, independently of preexisting hypogammaglobulinemia and possibly of treatment‐induced immunodepression. We also observed a drop in the serum response overtime and following autologous transplantation. Further studies in larger sample are needed to understand the benefit of vaccination strategies in these patients.

## 1 INTRODUCTION

Infections are a leading cause of morbidity and early mortality in multiple myeloma (MM) patients,^1^, ^2^, ^3^ often characterized with severe hypogammaglobulinemia, This is, in part, due to the increase in tumor plasma cells infiltrating the bone marrow and decrease in the percentage of normal gammaglobulin producing plasma.^4^, ^5^ Infections are a leading cause of morbidity and early mortality, with an estimated 50% of early deaths due to infection.^2^

*Streptococcus pneumoniae* is a common pathogen colonizing the upper respiratory tract that causes substantial morbidity and mortality from noninvasive diseases, such as otitis media and sinusitis, and invasive diseases, including pneumonia, septicemia, and meningitis.^1^ Pneumococcal infection is likely to be among the most severe infections that MM patients might encounter, although not the most frequent.^2^, ^6^ Indeed, pneumonia and septicemia are the most common serious diseases resulting from 66% and 23% of pneumococcal infections in myeloma, respectively.^2^

The risk of infections decreases in MM patients responding to treatment,^7^, ^8^ but increases again, when disease progression or relapse occurs.^7^ Current guidelines for individuals undergoing stem cell transplant recommend the use of pneumococcal vaccination and penicillin A prophylaxis to prevent pneumococcal infection^9^ and whether these treatments are beneficial to MM patients is currently unclear.

The two pneumococcal vaccines available for adults are the 13‐valent conjugated vaccine (Prevnar13®) and the 23‐valent polysaccharide vaccine (Pneumo23®; PPV). Prevnar® contains extracts from 13 serotypes conjugated to a protein carrier, whereas PPV contains extracts from 23 serotypes in their native polysaccharide structure. The former elicits a T‐cell–dependent immune response and is more immunogenic, as it is more effective in producing memory B cells. This response may be boosted further by subsequent vaccination with PPV.^10^ In immunocompromised patients, pneumococcal vaccinations are given as a prime‐boost (PB) schedule: Prevnar13® followed by Pneumo23® 8 weeks or more apart.^10^ However, there are no data available for MM patients to support such a strategy, or report the level of activity of antibodies post‐pneumococcal vaccination.

In this study, we investigated the longitudinal responses to pneumococcal vaccinations for four serotypes (IgG, IgG2, IgA and IgM) after vaccination according to the PB schedule in MM patients and correlate it with infection type and rate.

## 2 MATERIAL AND METHODS

### 2.1 Study objectives

The primary endpoint was the incidence rate of patients capable of obtaining an isotype response serum concentration after vaccination with two vaccines, according to Rose et al^11^ and Parker et al.^12^ Secondary endpoints included detailed isotype increase, time to first increase, further assessment of a decreased anti‐pneumococcal serum concentrations following treatment including autologous stem cell transplantation (ASCT), rate of infection with a special attention to pneumococcal infection.

We collected data on patients, myeloma, treatments, and adverse events of infectious type, with particular attention to pneumococcal infections. This study was conducted in accordance with “good clinical practice” and all applicable regulatory requirements, including the 2008 version of the Declaration of Helsinki. The study was approved by the human research committee of Lille.

### 2.2 Study population

Twenty‐eight patients diagnosed with myeloma^13^ were prospectively enrolled in this study from the hematology department of Lille, France. The median age was 66 years (range 44‐78) and the male to female ratio was 0.64. All patients, except one, presented with hypogammaglobulinemia during diagnosis (after exclusion of the M‐component); of whom, 21 (75%) had severe hypogammaglobulinemia < 4g/L. One patient (4%) presented with pneumococcal infection (pneumonia and septicemia) during diagnosis of myeloma. Fourteen patients had international staging system (ISS) score 1; 5 score 2; and 4 score 3^14^; according to the revised ISS,^15^ 3 patients had score 1; 13 score 2; and 3 score 3 (Table S1). Twenty‐five patients were newly diagnosed with MM, while two patients were studied at first relapse and one at second relapse. The induction regimens at the time of study entry contained systematically a proteasome inhibitor‐triplet‐based (n = 28); among them, 10 patients underwent ASCT (Table S2). Dexamethasone was given weekly at 40 mg oral route during induction and 12 patients with serious desease at the diagnosis had received 4 doses prior to induction as a block of 160mg.

### 2.3 Pneumococcal vaccination

All MM patients were vaccinated intramuscularly in the deltoid region with the two vaccines, Prevnar13® (Lederle Laboratories, Pearl River, NY, USA) and Pneumo23® (Pasteur Merieux MSD, Brussels, Belgium), according to the PB schedule, with Prevnar13® followed by Pneumo23®.^10^ Patients were allowed to receive cycles of induction chemotherapy in between the two vaccinations in order to receive the myeloma treatment.

Prevnar13® contains extracts of the following pneumococcal serotypes: 1, 3, 4, 5, 6A, 6B, 7F, 9V, 14, 18C, 19A, 19F, and 23F conjugated to Diphtheria CRM197 Protein. Pneumo23® contains extracts of the following unconjugated serotypes 1, 2, 3, 4, 5, 6B, 7F, 8, 9N, 9V, 10A, 11A, 12F, 14, 15B, 17F, 18C, 19F, 19A, 20, 22F, 23F, and 33F.

### 2.4 Patient vaccination

**Figure 1:**
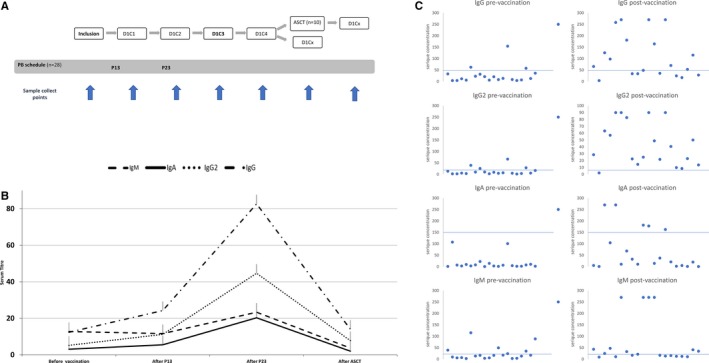
A, Longitudinal diagram of the vaccination schedule and the sample collect points. Each treatment cycle varied from 21 to 35 days depending of the age of the patient. D represent the day on the cycle and C the cycle number. B, Serum longitudinal profile of all patients. A slightly increase in all subtypes after P13 and a major increase after the P23, all immunoglobulin subtypes concentration decreased after ASCT. The last time point only concerns the eight patients whose Ig levels have been tested after ASCT. C, Anti‐pneumococcal serum concentrations of IgG, IgG2, IgM, and IgA for 20 patients before and after vaccination by P13 and P23. The blue bar represents the threshold antibody level correlated with a protection from invasive disease

Vaccination was given by a primary care physician with an average time of 30 days between the two injections and a wide variation (0‐119 days). The first sample was collected at the time of inclusion of the patient in the study before any vaccination and then sequentially at day 1 of each cycle of treatment (Figure 1A). The median time between the last vaccination and the first sample was 21 days (range 3‐91). The median time between pre‐ and postvaccination time point was 60.5 days (range 15‐202).

For this study, we separated patients according to the time from Prevnar13® to Pneumo23®; group 1 if the Pneumo23® (P23) occurs less than 30 days after the Prevnar13® (P13) and group 2 if the P23 occurs more than 30 days after P13.

### 2.5 Collection and ELISA test

Blood samples were drawn prior to vaccination and any treatment for all the patients, and then were sequentially collected at day 1 of each further cycle with an average of 6.14 sample per patient. These samples were centrifuged at 800 *g* for 10 minutes and the sera obtained were stored in aliquots frozen at −80°C until testing. The response to vaccination was obtained using the VaccZyme**™** pneumococcal capsular polysaccharide (PCP) IgG, IgG2, IgA, and IgM ELISAs for 23 serotypes (anti‐PCP23) (Binding Site Group Ltd, Birmingham, UK).

Anti‐PCP23 IgG, IgG2, IgM, and IgA were tested in 20 patients who fulfilled the inclusion criteria. Eight patients were not interpretable because of the absence of sampling before the vaccination or an early death before the second vaccination.

### 2.6 Statistical analysis

The vaccine response was studied according to IMWG myeloma response criteria.^16^ We regrouped the patients into two response categories: very good partial response (VGPR) and complete response (CR) altogether as great responders; and partial response (PR) and lower (stable disease [SD] and progressive disease [PD]) as lesser responders.

We used the IBM SPPSS statistic 23 software for statistical analysis. The increase in specific antibody concentrations (anti‐PCP23 IgG, IgG2, IgA, and IgM) was analyzed using the Wilcoxon matched pairs test for the 20 patients before and after vaccination. The increase in specific antibody concentrations (anti‐PCP23 IgG, IgG2, IgA, and IgM) was analyzed by the Wilcoxon matched pairs test for the 20 patients before and after vaccination according to the response to treatment. Comparison between good responders (CR or VGPR) and lesser responders (PR, SD, or PD) was analyzed by calculating the Ig‐fold for each patient and comparison between the two groups by the Mann‐Whitney Wilcoxon test. The decrease in specific antibody concentrations was analyzed by the Wilcoxon matched pairs test in 8 patients with MM before and after ASCT and in 15 patients after vaccination and several months later. Comparison between these two groups was analyzed by calculating the Ig‐fold for each patient and comparison between the two groups by the Mann‐Whitney Wilcoxon test. To determine responders and nonresponders, immunoglobulin subtypes concentrations were compared to published isotype‐specific antibody concentration.^11^, ^12^

## 3 RESULTS

### 3.1 Vaccine response

Among the 28 patients included and receiving the vaccinations, anti‐PCP23 data were available for 20 patients**.** Overall, anti‐pneumococcal serum concentrations of IgG, IgG2, IgM, and IgA increased by 5.1‐, 5.9‐ 1.5‐, and 3.8‐fold following anti‐pneumococcal vaccination, respectively (Table 1 and Figure 1B). Eight‐five percent (n = 17) of the patients obtained significant antibody concentration for at least one subtype, 65% (n = 13) for at least two subtypes, 55% (n = 11) for at least three and 2 patients responded to all four subtypes. Twelve patients (60%) had significant IgG antibody concentration, 15 patients (75%) had IgG2, 12 patients (60%) had IgM, and 5 patients (25%) had IgA. The threshold antibody level correlated with a protection from invasive disease is available in Rose et al^11^ for IgG and IgG2 and Parker et al^12^ for IgA and IgM: 51.5 µg/mL IgG, 19.5 µg/mL IgG2, l50 UI/mL IgA, and 20 U/mL IgM, respectively.

**Table 1:** Geometric mean concentration of anti‐PCP IgG, IgG2, IgA, and IgM in 20 patients with multiple myeloma pre‐ and postvaccination following two vaccines

Vaccine groups	Time to vaccination	IgG (mg/L)	IgG2 (mg/L)	IgA (U/mL)	IgM (U/mL)
P23 less than 30 days after P13	Pre				
	Median	16.50	7.52	7.32	7.81
	Min	3.92	2.11	0.48	2.23
	Max	62.26	39.05	107.2	115.04
	Post				
	Median	81.89	42.74	50.56	28.85
	Min	3.76	2	0.58	8.93
	Max	270	90	270	270
	*P*	0.007	0.013	0.037	0.009
P23 more than 30 days after P13	Pre				
	Median	13.26	5.82	3.57	17.18
	Min	4.19	1.30	1.08	2.73
	Max	154.38	66.37	100.64	88.51
	Post				
	Median	61.43	31.72	16.98	16.11
	Min	17.01	8.47	0.35	10.44
	Max	270	90	177.81	270
	*P*	0.013	0. 017	0.028	0.575

The anti‐pneumococcal serum concentration statistically increased for IgG, IgG2, and IgA, but not for IgM after PB vaccination (Table 1). This data shows that anti‐pneumococcal antibodies were detectable within 1 week after vaccination for the majority of patients (Figure 1B).

The anti‐pneumococcal serum concentration statistically increased for IgG, IgG2, and IgA, but not for IgM in the group 2, although it was statistically different for all four isotypes in the group 1 (Table 1).

### 3.2 Degree of disease control and pneumococcal vaccine response rate

Among the 20 patients included in this study, 5 reached CR, 7 VGPR, 4 PR, 3 SD, and 1 had PD. Our data showed that pneumococcal vaccine responses occurred independently of the degree of disease control, and thus did not correlate with the depth of response rate (Table 2).

**Table 2:** Geometric mean concentration of anti‐PCP IgG, IgG2, IgA, and IgM in 20 patients with multiple myeloma according to the disease control

Vaccine group	Time to vaccination	IgG (mg/L)	IgG2 (mg/L)	IgA (U/mL)	IgM (U/mL)
RC or VGPR (n = 12)	Pre				
	Median	13.26	5.86	7.15	11.2
	Min	4.14	1.3	0.48	3.42
	Max	57.65	28.42	107.2	35.06
	Post				
	Median	50.82	23.94	35.09	22.97
	Min	3.76	2	0.58	8.93
	Max	270	90	270	270
	*P*	0.006	0.012	0.028	0.05
RP, SD, or PD (n = 8)	Pre				
	Median	20.73	9.05	2.51	31.53
	Min	5.86	3.27	0.97	2.23
	Max	154.38	66.37	100.64	115.04
	Post				
	Median	117.06	44.66	12.26	25.01
	Min	17.01	8.47	0.35	10.42
	Max	270	90	162.41	270
	*P*	0.017	0.017	0.017	0.327
RC or VGPR vs RP, SD. or PD	*P*	0.34	0.73	0.85	1

### 3.3 Memory B lymphocyte immune response

We identified an increase in total IgG after the second vaccine dose illustrating the constitution of memory B lymphocytes following the first injection supporting the interest of this schedule (Figure 1B).

### 3.4 Loss of pneumococcal immunization with time

We compared anti‐pneumococcal IgG, IgG2, IgM, and IgA isotypes serum concentrations on a sequential manner. Most patients had samples collected prior to and sequentially after the pneumococcal vaccination; but for eight patients, samples were collected prior to, after the pneumococcal vaccination, and after the ASCT. Of note, the later eight patients got vaccinated with a median of 168.5 days (46‐233) from completion of PPV vaccination to ASCT. The median time between the prior and post‐ASCT serum samples was 278 days (range 154‐439) and was 115 days (68‐306) post‐ASCT. Results are summarized in Table 3. Interestingly, the anti‐pneumococcal geometric mean concentration decreased significantly for all subtypes over time independently of treatment approaches, although significantly more for patients that had ASCT on IgG and IgG2 (Table 3).

**Table 3:** Geometric mean concentration of anti‐PCP IgG, IgG2, IgA, and IgM in 8 patients with multiple myeloma pre‐ and post‐autologous stem cell transplantation and in 15 patients after vaccination and several months later

	IgG (mg/L)	IgG2 (mg/L)	IgA (U/mL)	IgM (U/mL)
ASCT				
Pre	110.5	52.74	26.32	28.8
Post	14	7.8	2.97	3.7
Fold decrease	7.9	6.8	12.9	7.7
*P*	0.008	0.008	0.008	0.008
No ASCT				
Postvaccination	95.47	48.76	32.77	34.57
Late postvaccination	34.34	17.10	4.13	8.48
Fold decrease	2.8	2.9	7.9	4.1
*P*	0.001	0.002	0.04	6.10 e‐5
ASCT vs no ASCT				
*P*	0.03	0.03	0.27	0.64

The serum concentrations decreased by 7.9‐fold for IgG, 9.8‐fold for IgG2, 7.7‐fold for IgM, and 1.3‐fold for IgA for patients who underwent ASCT (Figure 1B). Only two of them (25%) had one Ig subtypes with a level correlated with a protection from invasive disease, and only one (12.5%) for IgG. Six patients (75%) did not had any protective antibody level among the four subtypes.

Comparatively, we also observed a decrease in the anti‐pneumococcal geometric mean concentration for patients without ASCT in all subtypes, 2.8‐fold for IgG, 2.9‐fold for IgG2, 4.1‐fold for IgM, and 7.9‐fold for IgA, with a median follow‐up time of 224 days (28‐502). Among these patients, 60% had one Ig subtypes with a level correlated with a protection from invasive disease, only 20% for two subtypes, 7% for three subtypes, and only 26% for IgG.

### 3.5 Infections

Severe infection was observed in five (18%) patients, of whom two who died. No patient was presented with *S. pneumoniae* infection during the follow‐up neither identified on hemocultures, sputum culture, lumbar puncture, nor by urinary antigen assay.

## 4 DISCUSSION

Pneumococcal infection is a leading cause of morbidity and early mortality in patients with myeloma.^1^, ^2^, ^3^ A poor antibody vaccine response has been shown to be associated with an increased risk of septicemia in immunocompromised patients,^17^, ^18^ although an effective vaccination is a simple and effective way to decrease morbidity and mortality for these patients. However, myeloma is associated with severe hypogammaglobulinemia, and one could therefore anticipate impaired response to pneumococcal vaccinations. Interestingly, a primary care physician and/or hematologist routinely perform pneumococcal vaccine on the basis that myeloma patients are immunocompromised, but without data to support this supportive care approach. In this study, we sought to study the response to pneumococcal vaccination in MM patients, initially at diagnosis, overtime, and also to monitor the variations across treatment approaches.

We observed a significant increase in the geometric mean concentration of anti‐PCP IgG, IgG2, IgM, and IgA post‐pneumococcal vaccination in 20 MM patients, 80% of them obtained a protective antibody titer for at least one subtype including 60% for IgG, showing the ability of MM patients to respond after a pneumococcal vaccination. A part of this increase could also be explained from reduced immune suppression due to MM during treatment‐induced remission. However, a value over the threshold for one subtype does not necessarily prove an effective protection against any pneumococci, of which more than 90 serotypes have been identified so far.^19^, ^20^ The assessment of multi‐isotypes enable complete identification of a deficient antibody response. The ability to quantitate IgG1 and IgG2 subclasses is informative in understanding and evaluating the immune response(s) to PCP conjugate and nonconjugate vaccines. Adults have preexisting serotype‐specific IgG2 antibody, due to prior natural exposure to pneumococci, IgG1 and IgG2 responses may follow pneumococcal vaccine immunization for this population.^21^ After vaccination with both a protein‐based and a polysaccharidic vaccine, an antibody production responding to protein antigens is observed (IgG1 and IgG3 mainly) associate with an Ab production responding to polysaccharidic antigens (mainly IgG2) illustrated by the Figure 1C. The production of PCP IgM is pivotal for the protection against pneumococcal disease and has been shown to be defective in Common variable immunodeficiency (CVID).^22^ IgA and IgM are mainly used to evaluate the infectious risk for patients with immunologic deficit or receiving IVIG (no patient in the studied received intravenous immunoglobulin (IVIG) during the follow‐up). CVID patients with PCP IgA and IgM concentrations above 150 and 20 U/mL, respectively, have been shown to have a lower risk of developing pneumonia and bronchiectasis than those with either a PCP IgM concentration < 20 U/mL only or those with both concentrations below both the above cut‐offs.^22^

We have also observed a significant decrease in most patients overtime for all anti‐PCP median serum levels subtypes, and particularly after ASCT. This data tends to confirm that MM can respond to vaccination at diagnosis and relapse, but tend to lose this response overtime, especially if further immunocompromised with a myeloablative treatment approach.

As a comparison, P23 vaccination on patient without chronic disease^23^ report a trend of progressively decline of the postvaccination antibody level, similar to findings with other vaccines. However, in most of the studies, antibodies persisted for 3 or more years above the initial level or the level of unvaccinated adults.

Few studies have studied myeloma response to the vaccine with of 23‐valent PPV, and showed that 33‐40% responded to the vaccine with a low level of response as levels of anti‐pneumococcal antibodies were 15‐fold higher in the controls than in the MM group.^17^, ^24^, ^25^ Interestingly, we found a much greater rate of response with the combination of the two vaccines, Prevnar13® and Pneumo 23®, independently of the vaccination schema.

No pneumococcal infection was observed in our study after inclusion probably due to the number of patients included and limited longitudinal follow‐up (median of 224 days), especially in the ASCT group.

One patient presented with a pneumonia and further septicemia with *S. pneumoniae* was diagnosed for myeloma. This patient had not been vaccinated yet at time of this event as it was prior to MM diagnosis. This patient was then recruited into our study, was vaccinated, responded (increased for all subtypes by 1.3‐fold for IgG, 1.0‐fold for IgG2, 1.6‐fold for IgM, and 6.2‐fold for IgA) and never suffered any recurrence of *S. pneumoniae* infection since then. Although severe infection (septicemia) was reported in four other patients (after pneumococcal vaccination) with two co‐infections across treatment phases, none were of pneumococcal type. Indeed, one patient presented with *Staphylococcus haemolyticus* and *Enterococcus faecalis*, another with *Streptococcus oralis*, *Bacteroides fragilis*, and *Proteus mirabilis*, and two other with *Klebsiella pneumoniae *and *Streptococcus salivarius*. Four patients died during the follow‐up, two of them from infectious events. None of these germs is encapsulated.

The increased risk of infections is a well‐known and largely reported complication event of myeloma either at diagnosis or during the MM history course of the disease. Surprisingly, infection prophylaxis with vaccination, immunoglobulins, and antibiotics remains poorly investigated. One would recommend to vaccinate the patients against pneumococcus and to study the vaccine response, at least in order to protect the patients from occurrence of this likely severe infection; and also in order to stop antibioprophylaxis that has become common practice in many centers worldwide. Some questions remain: (a) this antibioprophylaxis could likely be recommended at time of the ASCT where we have demonstrated a significant drop in immune coverage protection. (b) One may hypothesize that the use of antibioprophylaxis (phenoxymethylpenicillin) plus pneumococcal vaccination may optimally help MM to prevent for *S. pneumoniae* infection. In our study, we have not initially planned to longitudinally study the vaccine response beyond ASCT, and therefore cannot anticipate whether or not the patients had spontaneously corrected the vaccine response serum titers drop or if a second vaccination would be recommended. A study with and without antibioprophylaxis in patients undergoing vaccination might help to optimize the patient’s supportive care regarding pneumococcal prophylaxis treatment approach.

There are now reliable tests to study and verify the vaccine response to pneumococcal vaccine in MM, but we believe the VaccZyme™ test used herein might be more appropriate given the current test objectives, for example, demonstrating the response to vaccine in MM patients. We have observed a rise in anti‐pneumococcal, across all Ig subtypes after vaccination, that improved the ability of MM to present with vaccine response. There was decrease in overtime and particularly a drop after ASCT, validating the test as able to demonstrate variation across treatments. This drop is in part due to the impact of the treatment especially ASCT but it could partially be explained by spontaneous decrease over the time as observed for all vaccines and majored by a more severe myeloma related immunosuppression in relapsing patients.

Interestingly, the anti‐PCP decrease was far more pronounced with IgG2, IgM, and IgA subtypes rather than IgG. We believe this data demonstrates that the conjugated vaccine use with high affinity elicited a T‐cell–dependent response, a class‐switched IgG1 and 3, and a memory response upon subsequent exposure to the same antigen in contrary to T‐cell–independent response that elicit low affinity IgM with IgG2 and IgA responses without memory response.

One of the limitations to our study is the small number of patients included due to the need to first validate this new test on a first studied cohort before the study could be enlarged and possibly performed on a more homogeneously treated population. Another limitation is the great variety in time points after vaccination and after ASCT. A study in a larger group of patients is therefore warranted to validate this data. Furthermore, follow‐up was insufficient to evaluate the evolution of the anti‐PCP response away from ASCT. Longer longitudinal follow‐up may be important for assessing anti‐pneumococcal immune reconstitution over time and especially after ASCT.

In conclusion, we demonstrated myeloma has the ability to demonstrate a response to pneumococcal vaccine, independently of preexisting hypogammaglobulinemia and disease response and possibly of treatment induced immunodepression, with an expected drop of response overtime and immediately following autologous transplantation. This response tends to be associated with protection against pneumococcal infection. Further studies in a larger group of patients is warranted to validate this data.

## CONFLICT OF INTEREST

The authors declare no potential conflicts of interest.

## AUTHOR CONTRIBUTIONS

X. L., L. R., and S. H. conceptualized and designed the study. L. R., G. F., S. G., H. D., M. N., E. C., C. B., C. H., T. S., A. M., F. S., A. L., A. B., V. R., N. M., C. G., D. D., Z. V. W., B. C., S. M., T. F., and X. L. conducted the study. S. S. and J. L. F. collection and assembled the data. X. L., L. R., S. H., and S. G. analyzed and interpreted the data. L. R., X. L., and S. H. wrote the manuscript.
