# Supplementary material for: Response to pneumococcal vaccination in multiple myeloma
Source: Cancer Med. 2019 May 30;8(8):3822–30. doi: 10.1002/cam4.2253 (PMC6639194; doi:10.1002/cam4.2253)
Supplement: Supplementary file 1 [file CAM4-8-3822-s001.docx]

**Supplement data Table 1**. Patients characteristics at study entry (n=28). Group 1 refers to patients who received Pneumo23® (P23) less than 30 days after the Prevnar13®(P13) and group 2 to patients who received the P23 more than 30days after P13.

| N (%), unless specified | group 1 | group 2 | Overall |
| --- | --- | --- | --- |
| Sex  Male  Female | 6  7 | 5  10 | 11  17 |
| Median age | 61 (44-76) | 68 (52 – 78) | 66 (44-78) |
| Gammaglobulin level*  Median  Range | 3.7  0,5-12,2 | 3  0,9-5,8 | 3  0,5 - 12,2 |
| ISS  I  2  3  UK | 5 (18%)  3 (11%)  2 (7%)  3 (11%) | 9 (32%)  2 (7%)  2 (7%)  2 (7%) | 14 (60%)  5 (18%)  4 (14%)  5 (18%) |
| Adverse cytogénétique  del(17p)  t(4;14) | 2 (7%)  1 (4%) | 1 (4%)  2 (7%) | 3 (11%)  3 (11%) |
| LDH  High | 3 | 8 | 11 (39%) |
| ISS-R  1  2  3  UK | 1 (4%)  6 (21%)  1 (4%)  5 (18%) | 2 (7%)  7 (25%)  2 (7%)  4 (14%) | 3 (11%)  13 (46%)  3 (11%)  9 (32%) |

*(excluding monoclonal peak); UK= unknown
