# Supplementary material for: Response to pneumococcal vaccination in multiple myeloma
Source: Cancer Med. 2019 May 30;8(8):3822–30. doi: 10.1002/cam4.2253 (PMC6639194; doi:10.1002/cam4.2253)
Supplement: Supplementary file 2 [file CAM4-8-3822-s002.docx]

**Supplement data Table 2**. type of treatments provided (n=28).

| Treatment provided | | Nb of patient |
| --- | --- | --- |
| ASCT | 11 (39%) | |
| PI triplet based | 28 (100%) | |
| Pi +alkylator | 10 (36%) | |
| Pi +IMiDs | 17 (60%) | |
| Pi + BCL2 inhibitor | 1 (4%) | |

ASCT: Autologous stem cell transplantation; PI: proteasome inhibitor; IMiDs: immunomodulatory Drugs
